# The effect of last meal “Suhoor” timing on diurnal variations in cognitive performance during Ramadan fasting among female athletes

**DOI:** 10.3389/fnut.2024.1373799

**Published:** 2024-04-17

**Authors:** Houda Bougrine, Achraf Ammar, Khaled Trabelsi, Ahlem Belgacem, Atef Salem, Hamdi Chtourou, Haitham Jahrami, Nizar Souissi

**Affiliations:** ^1^High Institute of Sport and Physical Education Gafsa, Gafsa University, Gafsa, Tunisia; ^2^Physical Activity Research Unit, Sport and Health (UR18JS01), National Observatory of Sports, Tunis, Tunisia; ^3^Department of Training and Movement Science, Institute of Sport Science, Johannes Gutenberg-University Mainz, Mainz, Germany; ^4^Research Laboratory, Molecular Bases of Human Pathology, LR19ES13, Faculty of Medicine of Sfax, University of Sfax, Sfax, Tunisia; ^5^High Institute of Sport and Physical Education Sfax, University of Sfax, Sfax, Tunisia; ^6^Interdisciplinary Laboratory in Neurosciences, Physiology, and Psychology: Physical Activity, Health, and Learning (LINP2), Faculty of Sport Sciences (UFR STAPS), Paris Lumières University (UPL), Paris Nanterre University, Nanterre, France; ^7^Research Laboratory, Education, Motricity, Sport and Health (EM2S), LR15JS01, High Institute of Sport and Physical Education of Sfax, University of Sfax, Sfax, Tunisia; ^8^Department of Psychiatry, College of Medicine and Medical Sciences, Arabian Gulf University, Manama, Bahrain; ^9^Government Hospitals, Manama, Bahrain; ^10^High Institute of Sport and Physical Education Ksar-Saïd, Manouba University, Mannouba, Tunisia

**Keywords:** Ramadan intermittent fasting, Suhoor timing, diurnal variation, nutrient timing, female athletes, sleep, cognitive performance

## Abstract

The present study aimed to investigate the effect of the timing of the last meal, “Suhoor”, on diurnal variation in cognitive performance during Ramadan intermittent fasting (RIF). In a randomized order, 26 adolescent female athletes (aged 15.9 ± 0.9 y) were tested in three sessions at 7:00 a.m., 12:00 p.m., and 5:00 p.m. across four different conditions: the 10 days preceding Ramadan (PR); the final 12 days of Ramadan (RO): two different conditions: Early Suhoor (S_Early_), and Late Suhoor (S_Late_); and, ultimately, the 10 days immediately after Ramadan (AR) with an in-between recovery period of ≥24 h. During each test session, the oral temperature (OT), simple reaction time test (SRT), choice reaction time test (CRT), attention test (ATT), and mental rotation test (MRT) were evaluated. The Pittsburgh Sleep Quality Index (PSQI) and daily diary intake were assessed across the three periods. Compared to PR, the sleep parameters assessed by the PSQI were significantly lower during the RO and AR periods. However, neither total dietary intake nor OT showed any changes due to RIF. Compared to PR, the afternoon performances of SRT, CRT, ATT, and MRT significantly declined under both the S_Early_ and S_Late_ conditions. Notably, midday performance decreased only during S_Late_, while morning performance remained unaffected in both conditions. Additionally, compared to S_Early_, these performances were better during S_Late_ in the afternoon and at midday. In summary, nutrient intake timing had a significant effect on the diurnal fluctuations in cognitive functions during Ramadan fasting, particularly around noon and in the afternoon. Our results illustrate the benefits of a late last meal (Suhoor) in preserving optimal morning cognitive abilities and preventing any impairment during the fasted state at midday or in the afternoon, which could affect overall athletic performance.

## 1 Introduction

In modern times, the indisputable influence of nutrition on an athlete's performance has become increasingly apparent, with a growing acknowledgment of its crucial role in cognitive function and mental wellbeing, particularly among diverse and physically active populations. In the last decades, there has been an emergence of various dietary patterns and protocols attempting to enhance the adaptations resulting from physical exercise to boost the athlete's performance (1, 2). A variety of diets most commonly examined among athletes include the Mediterranean diet, low-carb and ketogenic diets, vegetarian and plant-centric diets, and intermittent fasting (3). Intermittent fasting in particular has recently gained increased attention due to its purported impacts on health and enhancement of body composition in patients with prevalent contemporary pathologies (4). Intermittent fasting represents a favored dietary pattern that relies on designated periods of fasting and is adopted in various forms by diverse groups globally. In this context, epidemiological evidence suggests that diets, such as intermittent fasting, may play a crucial role in preventing brain-related diseases by influencing their interactions with the brain (5). Additionally, recent studies indicate that fasting periods, irrespective of changes in caloric or nutritional intake, can influence cognition and brain health, thus shifting attention toward the timing and frequency of meals alongside the established focus on dietary intake's impact on cognition (6, 7). However, fasting, the intentional abstention from food and drink for durations that can vary from a few hours daily to several weeks, lacks a clear definition for when it begins after the last intake of food or beverage (8, 9). Despite its increasing appeal, the impact of intermittent fasting on performance still appears ambiguous at present. This ambiguity holds particular significance when it comes to the physical and cognitive capabilities of athletes who often adopt this dietary regime or periodic caloric restrictions such as Ramadan intermittent fasting (9). During this month, Muslims athletes must observe a fast from food and liquids between dawn and sunset for a continuous 30-day period in the ninth month of the lunar calendar (10).

This fasting period causes a noticeable decline in both physical and cognitive performance, disrupts circadian rhythms, and affects endocrine and metabolic processes (10–12). Although complex interactions between mental and physical aspects characterize athletic performance, studies on athletes' cognitive abilities during this fasting period are limited compared to those involving physical exercise. In team ball sports, performance optimization involves a combination of physical, technical, tactical, and cognitive skills, and there is a growing acknowledgment among players of the importance of mental health and cognitive function in achieving success (13). Thus, specific recommendations for athletes in this field are crucial for optimizing athletic performance. Research on cognitive function has yielded inconsistent findings for people of various ages. Indeed, studies examining mood and psychomotor performance have reported, to varying extents, fast-induced changes in alertness and vigilance (10, 14); fatigue; drowsiness; and irritability (15, 16); as well as reaction time and memory (17, 18). Although recent studies concerning RIF have revealed improvements in specific cognitive functions tied to resilience, cognitive flexibility, and sustained speed of reaction, impairments in visuospatial memory and attention have also been indicated (19). The variations in the methods, durations of intermittent fasting, samples (age, sex, physical level), sleep quality, last meal timing, lifestyles, local culinary traditions, diets, nutritional status and customs could all be factors leading to the diversity of these findings (10, 20).

In addition, the consideration of the time of day is often overlooked, suggesting the importance of exploring the effects of various performance measures at different times. In addition to temporal factors, both exercise and nutrition emerge as potent factors that affect the structure and function of the brain (21). Even though there has been a recent acknowledgment of the importance of nutrient timing, the current guidelines lack consistency, especially during Ramadan fasting, due to limited evidence, which can be perplexing for athletes and coaches during this month. This lack of clear guidance on nutrient timing during fasting can potentially negatively affect performance and increase injury risk (22). Hence, it might be essential for athletes to adopt a personalized strategy for nutrient timing to maximize their performance (23). Surprisingly, limited investigations have focused on the optimal last meal timing “Suhoor” for athletes to maintain and/or improve their cognitive and physical performance during RIF. To our knowledge, only one recent study has investigated the optimal Suhoor timing for short-term high-intensity exercise (11), while no evidence has been found about the optimal Suhoor timing intake related to cognitive performance in athletes.

On the other hand, while sports studies have focused on macronutrients, meal timing, and hydration, they tend to overlook women's unique physiological factors impacting energy and fluid needs. Since metabolism and fluid retention in women are influenced by monthly hormonal oscillations as well as individual differences in size, body composition, and hormone profiles (24), special guidance on nutritional timing for female athletes is needed to achieve performance goals and minimize injury risk.

Taken together, despite the substantial research conducted on RIF over the past 70 years, there is still a need for additional data regarding how RIF affects cognitive abilities among athletes. Thus, gaining a deeper understanding of RIF will help improve its practice, maximize its health advantages, avoid injury risks, and provide guidance to healthcare providers when advising chronically active athletes about RIF-related concerns.

Owing to the scarcity of literature on the impact of RIF and the timing of the Suhoor intake on the diurnal variation in cognitive performance and sleep quality in young athletes, this study sought to assess the connection between the timing of the Suhoor intake and the daily variation in cognitive performance during RIF while controlling sleep quality and dietary intake. The study hypothesized that during Ramadan, (i) the diurnal fluctuations in cognitive performance and sleep patterns of Muslim female athletes may be disrupted, and (ii) consuming Suhoor later would have a more favorable impact on cognitive performance than consuming it earlier, with the most significant effect observed in afternoon performance.

## 2 Materials and methods

### 2.1 Participants

Following the recommended guidelines proposed by Beck (25), we used G^*^Power software (26) to determine the minimum necessary sample size in advance. The significance level (α) was established at 0.05, with a desired statistical power (β) of 0.80. After estimating effect sizes based on a previous study with a similar design (11) and after discussion among the authors, we approximated the effect size to be 0.25. To attain the requisite statistical power, it was determined that a sample size of at least 22 athletes would be sufficient, therefore minimizing the probability of a type 2 statistical error.

In total, 26 trained female adolescent team ball players (aged 15.9 ± 0.9 y, height: 1.70 ± 0 m, and body mass: 60.2 ± 6.6 kg) participated in this study to avoid dropping out during the experimental sessions. Before data collection, all participants' and participants' parents' consent was obtained. Ethical approval was obtained from the lead institution's research ethics board, which aligns with the guidelines of the Declaration of Helsinki (27). Athletes were included in the current study if they met the following inclusion criteria: (1) fasted for at least 4 years; (2) were between 15 and 20 years old; (3) participated in regular training (at least 2 training sessions per week) for at least 4 years; (4) did not report smoking, caffeine dependence, or daily habitual napping; (5) did not use any form of contraception, including pills, patches, injections, implants, or intrauterine devices; and/or had any menstrual or endocrine abnormalities in the previous 6 months; and (6) had no history of major medical conditions. The exclusion criteria were as follows: (1) had an extreme morning or evening chronotype, (2) had any sleep troubles, and (3) had an extreme Suhoor time habitually. To ensure uniformity and reduce the impact of participants' habitual Suhoor timing on our results, athletes were selected based on their Suhoor timing responses during the first 15 days of RIF. Those with extreme habitual Suhoor times (consumed at ≤10:30 ± 30 p.m. or ≥ 02:30 ± 30 a.m.) that aligned with the two chosen specified times in our study were excluded. Consequently, the study included participants with a daily habitual Suhoor timing of 01:30 ± 1 h a.m.

Based on Horne and Ostberg (28) questionnaire scores ranging from 46 to 57, all participants were classified as “neither type” chronotype. The athletes had an average sleep duration of 7.8 ± 0.8 h, as assessed using the Arabic version of the Pittsburgh Sleep Quality Index (PSQI) (29), in the month before the experiment. Since the participants' daily sleep-wake cycles were followed, they established a schedule before Ramadan involving regular meal timing (breakfast at 07:00 a.m. ± 1:00 h, lunch at 12:30 p.m. ± 1:00 h, and dinner at 08:30 a.m. ± 1:00 h) and regular sleeping habits (from 10:30 p.m. ± 1:00 h to 07:00 a.m. ± 1:00 h). Additionally, participants' menstrual cycle phases were tracked throughout testing sessions using the Mycalendar^®^ (Period Tracker) mobile application (30).

### 2.2 Experimental design

**Figure 1:**
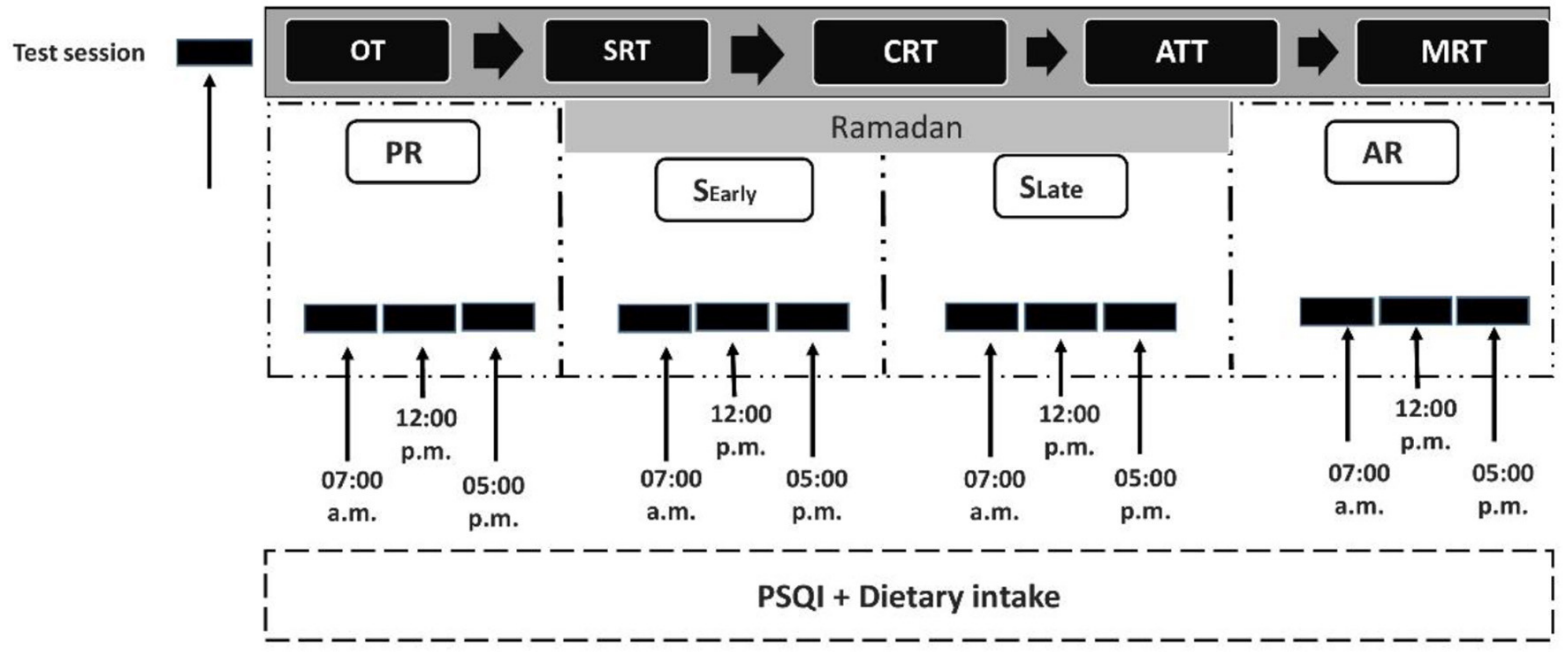
Study design. PR: the 10 days preceding Ramadan, S_Early:_ Early Suhoor condition during the final 12 days of Ramadan, S_Late:_ Late Suhoor condition during the final 12 days of Ramadan, AR: the 10 days right after Ramadan, PSQI, Pittsburgh Sleep Quality Index; OT, Oral temperature; SRT, Simple reaction time; CRT, Choice reaction time; MRT, Mental rotation test, all times given are expressed in local time (GMT + 1 h).

A randomized counterbalanced crossover study involved all participants going through testing on four different occasions: the 10 days preceding Ramadan (PR), the final 12 days of Ramadan (RO), with two different conditions: Early Suhoor (S Early), and Late Suhoor (S Late), and, ultimately, the 10 days immediately after Ramadan (AR). During each of these periods, players underwent three counterbalanced identical experimental trials, with only a single test session per day. These sessions took place in the morning (07:00–08:00 a.m.), at midday (12:00–01:00 p.m.), and in the afternoon (05:00–06:00 p.m.), separated by at least 24 hours between each session. These specific times were chosen because they corresponded approximately to the phases of highest oral temperature (31, 32) and because of better cognitive performance for this chronotype (neither chronotype) and because they were positioned during lunchtime midway between two reference times (Figure 1).

In the 2 weeks before the study, participants completed three familiarization sessions at 7:00 a.m., 12:00 p.m., and 5:00 p.m. to ensure reliable results and reduce learning effects. Participants were instructed to refrain from consuming any caffeine-containing substances and engaging in strenuous physical activity the day before the test and the day of the test. The athletes exercised once a day, from 8:30 p.m. ± 30 min to 9:30 p.m. ± 30 min during the entire Ramadan month. The participants were instructed to eat the same meals as they did the day before each trial. For the experimental sessions, players arrived at their regular training location, an indoor court, 1 h before the sessions began to collect the data and basic anthropometric measurements and perform the experimental trials. At the outset of each experimental session, oral temperature and anthropometric data were recorded. Body mass was measured using an electronic scale (Tanita, Tokyo, Japan) during morning sessions across all testing periods. Participants were advised to defecate and urinate before body mass was measured (33). A thermometer (Omron, Paris, France; accuracy +0.05°C) was placed under the tongue for at least 3 min following a 10-min resting period while the participants were seated to measure oral temperature.

Afterward, they underwent a battery of cognitive performance tests, which consisted of the simple reaction time test (SRT), choice reaction time test (CRT), attention test (ATT), and mental rotation test (MRT), in the same order. A 5-min rest period was used between these tests to ensure proper recovery (Figure 1). Athletes were only allowed to leave when the test was appropriately finished. Furthermore, daily caloric consumption and sleep quality were evaluated throughout each of the three periods.

Throughout the three experimental periods of our investigation, PR, RO, and AR, the testing sessions were held at an indoor training facility with consistent ambient temperatures (~25°C, 27°C, and 28°C) and relative humidity levels (~54%, 48%, and 49%, respectively). This investigation was conducted in Tunisia, coinciding with Ramadan, from April 2nd to May 1st, 2022. The fasting periods spanned ~15–16 h each day, occurring from ~04:25 a.m. to 07:10 p.m. local time.

#### 2.2.1 Suhoor protocol

Players experienced two distinct Suhoor conditions randomly throughout the Ramadan period: (S_Early_) required ingesting an Early Suhoor at 10:30 p.m., while (S_Late_) implied consuming a Late Suhoor at 2:30 a.m. During testing days, participants ingested two meals: the initial one at iftar (occurring between 7:00 p.m. and 7:30 p.m.), and the subsequent one between 10:30 p.m. and 11:30 p.m. on days 18, 20, and 22 to guarantee an early Suhour; between 2:30 a.m. and 3:30 a.m. on days 24, 26, and 28 to guarantee a late Suhour; or at any one of these two times on the days that persisted.

Every athlete received the same standard Suhoor, which included 0.5 L of water and ~700–750 kcal, distributed between 60% carbohydrate, 20% protein, and 20% fat. These dietary recommendations were established based on the results of food diary analysis and evaluation by the same nutritionist before and during Ramadan. The timing of the meals was the main distinction between the athletes' standard diets and the Suhoor meals in terms of macronutrient composition. It is crucial to remember that the athletes underwent rigorous observation throughout both trials, and an investigator confirmed their meal consumption. Before going to bed, participants abstained from eating for at least 20 min. The direction that was provided was restricted to the timing of Suhoor and did not include any particular instructions regarding the duration of sleep. After this standard meal, participants were not permitted to eat or drink anything at all. The instructions for the Suhoor meal were presented in the same, consistent way each time the subject requested administration. The assessments' day and timing for Suhoor and tests were announced previously.

#### 2.2.2 Dietary intake

Each participant was instructed to record all the amounts and varieties of food they consumed over the course of 10 consecutive days. The athletes were advised to provide nutritional information and, if applicable, the product's brand name. All the food-related diaries were checked as they were being collected. This was followed by an interview with a qualified dietician. The same dietician used the NUTRISOFT-BILNUT (version 2.01; Paris, France) computerized nutritional system to evaluate dietary records using food composition tables from the National Institute of Statistics of Tunis (1978)^1^ and the Bilnut program (Nutrisoft, Cerelles, France).

#### 2.2.3 Sleep assessment

The PSQI score ranges from 0 to 21, with 0 indicating no difficulty and 21 indicating serious issues with sleep parameters (34). There are 19 items in this questionnaire that focus on seven distinct components of sleep, including sleep quality, sleep duration, sleep latency, sleep efficiency, sleep disturbances, daytime dysfunction, and the use of sleeping medications. The scores for each of the seven components are determined using a score whose values range from 0 to 3. The final score of this written questionnaire was obtained by adding the outcomes of each of its seven components. Poor and very poor sleep quality are indicated by PSQI scores ≥ 5 and ≥ 8, respectively.

#### 2.2.4 Simple reaction time (SRT)

The processing speed was measured by evaluating the simple reaction time (SRT). Participants were given directions to rapidly press a button once a visual cue became visible on a computer display. The simple reaction time (SRT) test was administered using Reaction, INRP-free software (version 4.05) designed by Tilquin.

#### 2.2.5 Choice reaction time (CRT)

Using the same reaction, INRP software was used to display a colored geometric form referred to as the “target”. Subsequently, a series of variously colored geometric forms appeared on the screen. Whenever the target appeared, participants were instructed to press a button as quickly as they could. The software measured the time elapsed between the appearance of the form and the participant's response in seconds, with higher scores indicating less favorable performance.

#### 2.2.6 Attention test (ATT)

The digit cancellation test, also known as the attention test, is a valuable tool for evaluating cognitive-perceptual motor function, psychomotor speed, and sustained attention and vigilance (35). Its purpose is to assess various aspects of the prefrontal cortex's functioning, including the ability to focus attention, information processing speed, and executive functioning (36). Participants performed the test by crossing out target numbers (i.e., numbers composed of three digits) on a sheet of randomly arranged numbers. The participant's attention was evaluated based on the number of correctly detected targets, with one point awarded for each correctly identified 3-digit number in 1 min.

#### 2.2.7 Mental rotation test (MRT)

The Mental Rotation Test (MRT) was also conducted using Open Sesame software version 3.1 (37). In this test, participants are tasked with determining if two stimuli displayed on the screen are identical, following the principles of Shepard and Metzler (38). Each set in the test consisted of 10 items and required mental concentration, precision, and speedy manipulation. The test yields two types of results: the time taken to process correct responses (MRT_time_) and the number of errors made (MRT_errors_).

### 2.3 Statistical analysis

STATISTICA 10 software (StatSoft, Paris, France) was used to process all the statistical tests. For each variable, the means ± SDs (standard deviations) were determined. All the data were normally distributed, as confirmed by the Shapiro–Wilk test. Two-way repeated-measures ANOVA [4 (testing conditions) × 3 (time of day)] was also conducted to analyze the effects of the timing and duration of the day. One-way repeated measures analysis of variance (ANOVA) with three testing periods (PR, RO, and AR) was used to analyze the data, including PSQI score, body mass, BMI, total energy intake (kcal/day), fat intake (kcal/day), carbohydrate intake (kcal/day), and protein intake (kcal/day). Where appropriate, significant differences between means were tested using Tukey's HSD *post hoc* test. The magnitude of the difference between age groups was assessed using the effect size statistic (ηp^2^). The criteria used to determine the effect sizes were as follows: 0.01 denoted a small effect size, 0.06 represented a moderate effect size, and 0.14 indicated a large effect size (39). Standardized effect size (Cohen's d) analysis was used to interpret the magnitude of differences between variables, which were classified according to Hopkins (40) as trivial (d ≤ 0.20), small (0.20 < d ≤ 0.60), moderate (0.60 < d ≤ 1.20), large (1.20 < d ≤ 2.0), very large (2.0 < d ≤ 4.0), or extremely large (d > 4.0). A *p* value ≤ 0.05 was considered to indicate statistical significance.

## 3 Results

### 3.1 Daily caloric intake and body composition

Measures related to dietary intake, body mass (BM), and body mass index (BMI) across the three testing periods are summarized in Table 1. One-way ANOVA revealed significant main effects on daily carbohydrate intake [F_(2,50)_ = 5.84, *p* < 0.01, ηp^2^ = 0.18]. However, no significant variations were detected in protein [F_(2,50)_ = 1.20, *p* = 0.30, ηp^2^ = 0.04], fat [F_(2,50)_ = 0.69, *p* < 0.50, ηp^2^ = 0.02], or total caloric intake [F_(2,50)_ = 2.80, *p* = 0.06, ηp^2^ = 0.10] across the three testing periods. Further analysis indicated a reduction in daily caloric carbohydrates only during RO (*p* < 0.01) compared to PR (Table 1). In terms of body composition, there was a significant main effect of BM [F_(2,50)_ = 3.33, *p* < 0.05, ηp^2^ = 0.11] and BMI [F_(2,50)_ = 3.22, *p* < 0.05, ηp^2^ = 0.11]. Both BM and BMI decreased only during RO (both *p* < 0.05) compared to PR (Table 1).

**Table 1:** Measurement of the subjective sleep parameters, body composition, and daily caloric intake recorded during the 10 days preceding Ramadan (PR), the final 12 days of Ramadan (RO), and the 10 days right after Ramadan (AR).

		**PR**	**RO**	**AR**
Body composition	Body mass (kg)	60.2 ± 6.6^**b**^	59.8 ± 6.4^**a**^	59.9 ± 6.4
	BMI (kg/m^2^)	21.8 ± 2.1^**b**^	21.7 ± 2.1^**a**^	21.8 ± 2
PSQI	Total score PSQI (au)	2.2 ± 0.9^**bb, cc**^	7.3 ± 1.4^**aa, cc**^	5.1 ± 2^**aa, bb**^
	Sleep quality (au)	0.4 ± 0.3^**bb, cc**^	2 ± 0.6^**aa, cc**^	1.2 ± 0.5^**aa, bb**^
	Sleep duration (h)	7.8 ± 0.8^**bb, cc**^	6 ± 0.9^**aa, cc**^	7.2 ± 0.7^**aa, bb**^
	Sleep latency (min)	15.7 ± 2.1	16.1 ± 2.3	16.3 ± 2.6
	Sleep efficiency (%)	97.7 ± 2^**bb**^	94.5 ± 4.8^**aa**^	96.1 ± 4.1
	Sleep disturbance (au)	0.36 ± 0.2^**bb, cc**^	1.46 ± 0.4^**aa, cc**^	0.94 ± 0.4^**aa, bb**^
	Day time dysfunction (au)	0.2 ± 0.2^**bb, cc**^	1.3 ± 0.5^**aa, cc**^	0.7 ± 0.4^**aa, bb**^
	Use of sleep medication (au)	0 ± 0.02	0.01 ± 0.03	0 ± 0
Dietary intake	Carbohydrate (Kcal/d)	1,491.1 ± 204^**bb**^	1,447.1 ± 180.2^aa^	1,474.8 ± 190.9
	Protein (Kcal/d)	236.3 ± 50.8	230.3 ± 60.8	241.7 ± 56.4
	Fat (K/d)	732.46 ± 104.03	725.88 ± 114.29	721 ± 97.5
	Total energy intake (kcal/day)	2,453.3 ± 273.7	2,409.8 ± 249.4	2,437.5 ± 261.9

^**a**^*p* < 0.05, ^**aa**^*p* < 0.01: Significant difference compared to PR. ^**b**^*p* < 0.05, ^**bb**^*p* < 0.001: Significant difference compared to RO. ^**cc**^*p* < 0.001: Significant difference compared to AR.

### 3.2 Sleep assessment

Table 1 illustrates the variation in the subjective sleep parameters across the three testing periods (PR, RO, and AR). The statistical analysis revealed significant main effects of various aspects of the PSQI questionnaire, including total score [F_(2,50)_ = 96.18, *p* < 0.001, ηp^2^ = 0.79], sleep duration (F_(2,50)_ = 71.43, *p* < 0.001, ηp^2^ = 0.74], sleep quality [F_(2,50)_ = 128.25, *p* < 0.001, ηp^2^ = 0.84], sleep disturbances [F_(2,50)_ = 9.71, *p* < 0.001, ηp^2^ = 0.27], and daytime dysfunction [F_(2,50)_ = 47.24, *p* < 0.001, ηp^2^ = 0.65]. However, no significant variations were observed in sleep latency [F_(2,50)_ = 2.97, p = 0.06, ηp^2^ = 0.10] or the use of sleeping medication [F_(2,50)_ = 0.79, p = 0.45, ηp^2^ = 0.03] across the three testing periods. The Tukey test demonstrated that sleep duration was significantly shorter during RO and AR than during PR (both *p* < 0.001), and sleep efficiency was significantly reduced only during RO (*p* < 0.001). Furthermore, sleep quality, sleep disturbance, daytime dysfunction, and total PSQI score were significantly higher during both RO (all *p* < 0.001) and AR (all *p* < 0.001) than during PR (Table 1).

### 3.3 Oral temperature

There were no significant effects for COD [F_(3,75)_ = 1.5, p = 0.21, ηp^2^ = 0.2] or for the COD × TOD interaction [F_(6,150)_ = 0.7, p = 0.66, ηp^2^ = 0.02]. However, there was a significant main effect for TOD [F_(2,50)_ = 192.1, *p* < 0.001, ηp^2^ = 0.88], indicating that the core temperature was significantly greater at 5:00 p.m. Compared with that at 07:00 a.m. and 12:00 p.m., in all four testing conditions (all *p* < 0.001), the core temperature had an amplitude of morning-afternoon differences of 2.2%, 2.1%, and 2.1% during the PR, S_Early_, S_Late_, and AR (Table 2), respectively.

**Table 2:** Values (mean ± SD) of oral temperature, simple reaction time (SRT), choice reaction time (CRT), attention test (ATT), and mental rotation test (MRT) scores registered during the three times of the day (07:00 a.m., 12:00 p.m., and 5:00 p.m.) across four testing conditions: the 10 days preceding Ramadan (PR), the final 12 days of Ramadan (RO) with two different conditions: Early Suhoor (S_Early_), and Late Suhoor (S_Late_), and the 10 days right after Ramadan (AR).

	**Time of day**	**PR**	**S_Early_**	**S_Late_**	**AR**
Oral temperature (°C)	7:00 a.m.	36.05 ± 0.24^*^	36.03 ± 0.26^*^	36.05 ± 0.27^*^	36.06 ± 0.27^*^
	12:00 p.m.	36.30 ± 0.35^*, #^	36.27 ± 0.37^*, #^	36.33 ± 0.36^*, #^	36.30 ± 0.36^*, #^
	5:00 p.m.	36.85 ± 0.32	36.79 ± 0.28	36.81 ± 0.26	36.81 ± 0.28
SRT (ms)	7:00 a.m.	335.65 ± 15.02^*^	338.08 ± 14.81^*^	336.88 ± 14.46^*^	336.81 ± 15.60^*^
	12:00 p.m.	359.15 ± 17.37^*, #, **bbb, c**^	379.92 ± 19.62^**#, aaa, ccc**^	366.04 ± 17.18^**#, a, bbb**^	360.92 ± 17.55^*, **#**, **bbb**^
	5:00 p.m.	316.73 ± 15.84^**bbb, ccc**^	383.31 ± 14.45^**aaa, ccc**^	367.38 ± 13.99^**aaa, bbb**^	323.77 ± 15.81^**a, bbb, ccc**^
CRT (ms)	7:00 a.m.	456.19 ± 40.11^*^	458.15 ± 40.71^*^	457.65 ± 39.57^*^	456.69 ± 40.34^*^
	12:00 p.m.	484.85 ± 36.70^*, #, bbb^	501.50 ± 37.90^*, #, aaa, ccc^	488.92 ± 36.57^**#, bbb**^	485.46 ± 36.01^*, #, bbb^
	5:00 p.m.	431.62 ± 49.00^**bbb, ccc**^	513.58 ± 49.92^**aaa, ccc**^	490.65 ± 45.89^**aaa, bbb**^	441.12 ± 46.69^**a, bbb, ccc**^
ATT (au)	7:00 a.m.	63.81 ± 4.79^*^	62.92 ± 5.38^*^	63.04 ± 5.23^*^	63.58 ± 4.84^*^
	12:00 p.m.	61.08 ± 4.59^*, #, bbb^	58.69 ± 4.70^**#, aaa, c**^	60.15 ± 4.76^*, #, b^	61.00 ± 4.66^*, #, bbb^
	5:00 p.m.	68.35 ± 5.18^**bbb, ccc**^	58.58 ± 6.09^**aaa, ccc**^	65.73 ± 4.94^**aaa, bbb**^	67.04 ± 4.90^**bbb**^
MRT _time_ (s)	7:00 a.m.	2.38 ± 0.23^*^	2.39 ± 0.24^*^	2.40 ± 0.25^*^	2.40 ± 0.25^*^
	12:00 p.m.	2.60 ± 0.25^*, #^	2.63 ± 0.30^*, #^	2.62 ± 0.29^*, #^	2.61 ± 0.27^*, #^
	5:00 p.m.	1.99 ± 0.21	2.01 ± 0.23	2.003 ± 0.22	1.99 ± 0.22
MRT _errors_ (au)	7:00 a.m.	4.50 ± 0.58^*^	4.65 ± 0.75^*^	4.62 ± 0.75^**^	4.54 ± 0.51
	12:00 p.m.	4.96 ± 0.60^*, ##, bb^	5.46 ± 0.81^**#, aa**^	5.08 ± 0.69^**##**^	5.00 ± 0.69^*, ##, b^
	5:00 p.m.	3.92 ± 0.56^**bbb, ccc**^	5.73 ± 0.78^**aaa, ccc**^	5.04 ± 0.66^**aaa, bbb**^	4.19 ± 0.69^**bbb, ccc**^

^*^p < 0.001, ^**^p < 0.05: Significant difference compared to 5:00 p.m. (in the same condition); ^#^*p* < 0.001, ^##^*p* < 0.05: Significant difference compared to 7:00 a.m. (in the same condition); ^**a**^*p* < 0.05, ^**aa**^*p* < 0.01, ^**aaa**^*p* < 0.001: Significant difference compared to PR; ^**b**^*p* < 0.05, ^**bb**^*p* < 0.01, ^**bbb**^*p* < 0.001: Significant difference compared to S_Early_; ^**c**^*p* < 0.05, ^**ccc**^*p* < 0.001: Significant difference compared to S_Late_.

### 3.4 Simple reaction time (SRT)

There was a significant effect of TOD [F_(2,50)_ = 57.07, *p* < 0.001; ηp^2^ = 0.69], COD [F_(3,75)_ = 226.77, *p* < 0.001; ηp^2^ = 0.90], and the COD × TOD interaction [F_(6,150)_ = 139.09, *p* < 0.001; ηp^2^ = 0.84] on SRT. The *post hoc* test demonstrated an increase in SRT performance from 07:00 a.m. to 5:00 p.m. (both *p* < 0.001), with a decrease at midday (both *p* < 0.001) before Ramadan and before AR. However, the daily morning-afternoon differences were reversed during S_Early_ (*p* < 0.001) and S_Late_ (*p* < 0.001) (Table 2).

Regarding the effects of Suhoor scheduling, in comparison with PR, there was a significant decrease in SRT performance during SEarly (*p* < 0.001, 21%) and SLate (*p* < 0.001, 16%) in the afternoon and at midday (*p* < 0.001, 5.8%) and (*p* < 0.05, 1.9%), respectively. However, there were no significant changes between the two Suhoor conditions reported in the morning (both *p* > 0.05) compared to those in the PR. Additionally, SRT was significantly lower during S_Early_ than during S_Late_ in both the afternoon and midday sessions (both *p* < 0.001) (Table 2).

### 3.5 Choice reaction time (CRT)

Statistical analysis of CRT revealed significant main effects of TOD [F_(2,50)_ = 38,49; *p* < 0.001; ηp^2^ = 0,60], COD [F_(3,75)_ = 181,37; *p* < 0.001; ηp^2^ = 0,87] and the COD × TOD interaction [F_(6,150)_ = 115,69; *p* < 0.001; ηp^2^ = 0,82]. Compared to that in the morning, CRT performance was better in the afternoon (both *p* < 0.001) and lower at midday (both *p* < 0.001) before Ramadan and during the AR, respectively. However, these diurnal variations were reversed during S_Early_ (*p* < 0.001) and S_Late_ (*p* < 0.001), with better performance recorded during morning sessions (Table 2).

Regarding the effects of the Suhoor timing, compared to those of the PR timing, there was a significant decline in CRT performance during the S_Early_ (*p* < 0.001, 19%) and S_Late_ (*p* < 0.001, 13.5%) periods in the afternoon and only during the S_Early_ (*p* < 0.001, 3.4%) period at midday. In contrast, morning CRT performance did not significantly differ between the two suhoor conditions and the PR condition (both *p* > 0.05). Furthermore, in both the afternoon and at noon, CRT was significantly lower during the S_Early_ session than during the S_Late_ session (both *p* < 0.001) (Table 2).

### 3.6 Attention test (ATT)

Two-way ANOVA demonstrated a significant main effect of TOD [F_(2,50)_ = 26,28; *p* < 0.001; ηp^2^ = 0,51] and COD [F_(3,75)_ = 98,71; *p* < 0.001; ηp^2^ = 0,79] on ATT. Moreover, a significant COD × TOD interaction [F_(6,150)_ = 52,16; *p* < 0.001; ηp^2^ = 0,67] was reported. The *post hoc* test indicated that for PR and AR, ATT scores were greater in the afternoon than in the morning, with an increase observed at lunchtime (both *p* < 0.001 compared to the morning). Further analyses indicated that these daily variations were reversed during S_Early_ (*p* < 0.001) and blunted during S_Late_ (*p* < 0.01) (Table 2).

In terms of the Suhoor timing effects, ATT performance significantly declined during S_Early_ (*p* < 0.001, −14.3%) and S_Late_ (*p* < 0.001,−3.8%) in the afternoon and only during S_Early_ (*p* < 0.001, −3.9%) at midday compared to PR. On the other hand, the morning ATT scores did not change in comparison to those of the PR under either of the two Suhoor conditions (both *p* > 0.05). Additionally, in both the afternoon (*p* < 0.001) and noon (*p* < 0.05) sessions, ATT was significantly better during S_Late_ than during S_Early_ (Table 2).

### 3.7 Mental rotation test (MRT)

#### 3.7.1 MRT_time_

ANOVA did not reveal a significant main effect for COD [F_(3,75)_ = 2,57; p = 0.06; ηp^2^ = 0,09] or for the COD × TOD interaction [F_(6,150)_ = 0,92; p = 0.48; ηp^2^ = 0,03]. However, a significant main effect of TOD [F_(2,50)_ = 166,25; *p* < 0.001; ηp^2^ = 0,86] was observed. The MRT_time_ was lower at 07:00 a.m. than at 5:00 p.m. (all *p* < 0.001), and the MRT time decreased at midday (all *p* < 0.001) during all testing conditions (Table 2).

#### 3.7.2 MRT_errors_

TOD [F_(2,50)_ = 11.22, *p* < 0.001, ηp^2^ = 0.30] and COD [F_(3,75)_ = 46.51, *p* < 0.001, ηp^2^ = 0.65] had significant effects on MRTerrors. In addition, there was a significant COD × TOD interaction [F_(6,150)_ = 20.66, *p* < 0.001, ηp^2^ = 0.45], indicating that the diurnal variation in MRTerrors depends on Suhoor conditions. The *post hoc* analysis indicated that, compared to those in the morning, the MRT errors in the afternoon were lower during the PR (*p* < 0.001), and the number of errors increased at midday (*p* < 0.05 compared to the morning). However, the daily morning-afternoon differences were reversed during both S_Early_ (*p* < 0.001) and S_Late_ (*p* < 0.05) but disappeared during AR (*p* > 0.05) (Table 2).

Regarding the impact of Suhoor timing, compared to PR, there was a significant increase in MRT errors during S_Early_ (*p* < 0.001, 46.1%) and S_Late_ (*p* < 0.001, 28.4%) in the afternoon and only during S_Early_ (*p* < 0.01, 10.1%) in the midday. However, there were no significant changes between the two Suhoor conditions reported in the morning (both *p* > 0.05) compared to those in the PR. Additionally, the MRT errors were significantly higher during S_Early_ than during S_Late_ only in the afternoon (*p* < 0.001) (Table 2).

## 4 Discussion

Adopting a multifactorial methodology to investigate the various variables that could impact enhancing performance during Ramadan month, this is the first study to evaluate the association between Suhoor timing during RIF and the diurnal variation in cognitive performance (three-time of day) while controlling for sleep patterns and dietary intake among female athletes. We hypothesized that while fasting, the timing of the Suhoor might interfere with the diurnal variation in cognitive function of fasters, specifically, their attempts to exercise cognitive control with an optimal late-time intake. The key findings from this study reported that increasing the duration of the last meal intake was associated with a decline in cognitive performance in female players. Furthermore, shorter sleep duration and quality and unchanged total daily energy intake were associated with RIF.

### 4.1 Diurnal variations

The assessment of cognitive performance (SRT, CRT, ATT, MRT_time_, and MRT_errors_) indicated improved results during afternoon sessions compared to the two other times of day (morning and midday) before Ramadan. Cognitive performance seems to be time of day dependent, with significant increasing values from morning to afternoon, followed by a decrease during noon sessions. These findings are in agreement with previous studies revealing that time of day and task type could impact cognitive performance, with better performance recorded during afternoon sessions (41, 42) for attention (43, 44) and memory (45), executive control and processing speed (24, 46), vigilance (10, 24, 46), and executive functions (47). Conversely, other studies (48, 49) have reported that cognitive performance is better in the morning than in the afternoon. Pahan and Singh (50) also reported that cognitive functioning in preadolescent athletes was unaffected by the time of day. These discrepancies may be attributed to factors such as chronotype (neither chronotype in our study), age (adolescent in our study), sex, and participant wake-up time (51).

Regarding the effect of Ramadan on daily variation, limited research has been conducted on the influence of Ramadan Intermittent Fasting (RIF) on daily fluctuations in cognitive performance (10, 44). However, our data revealed that this daily fluctuation in cognitive performance was affected by RIF. The daily variations in SRT, CRT, ATT, and MRT_errors_ were reversed and/or blunted and/or disappeared toward the end of Ramadan month, mainly due to a decrease in afternoon performance. These findings are consistent with those of Bougrine et al. (10) and Khemila et al. (44), who reported a decrease or reversal in the diurnal variation of cognitive performance, such as SRT and attention tasks during Ramadan, primarily due to impaired afternoon performance.

#### 4.1.1 RIF effects

In the context of Ramadan's impact, there was a reduction in cognitive performance only during the afternoon, regardless of the presence of the Suhoor condition (10). However, this decline was not observed in the morning compared to before Ramadan (PR) or after Ramadan (AR). These results align with previous findings that have shown a negative effect of RIF on reaction time processing speed (18, 44), attention (10, 44, 52), vigilance (18) and simple reaction time (10, 44). However, other studies have not identified any alterations in cognitive abilities during RIF in terms of simple reaction time (53, 54), choice reaction time (54, 55), vigilance (56), or attention (52, 57, 58). Interestingly, studies that found no alterations in cognitive performance during RIF often did not control for or suggest an overall significant negative effect on sleep parameters.

In the present investigation, we suggest that impaired cognitive performance during RIF is strongly linked to sleep disruption and nutritional scheduling. RIF frequently causes sleep disturbances, which may contribute to a deterioration in cognitive function (59). Our results reveal a significant reduction in subjective sleep duration and quality during RIF, aligning with studies that associate cognitive decline with cumulative sleep deprivation and fragmentation throughout this month (10, 11). Considering athletes may require more than the standard 7–9 h of sleep for optimal performance (60) and that partial sleep deprivation is known to compromise athletes' cognitive abilities, the decline in cognitive function observed in our study during the afternoon could partly be attributed to this phenomenon (61, 62).

Fasting practices, such as Ramadan (Muslims) or Yom Kippur (Jewish faster), can have effects comparable to those of hunger, including headaches and dehydration (63, 64). Increasing attention to food (65) and dehydration (66) can have an impact on fasters' cognitive abilities even when they are not hungry.

The extended fasting duration in our study (15–16 h) can be mainly responsible for the observed performance drop, especially in the afternoon, regardless of Suhoor timing. Athletes can experience fatigue and decreased performance during RIF (67) due to lower blood glucose levels, decreased glycogen stores, and decreased overall energy availability, especially 6 h after the last meal (68). Moreover, changes in sleep and eating habits caused by RIF may disrupt circadian rhythms, affect metabolism (16, 69), and cause stress (16, 70). This can alter athletes' mindset motivation and cognitive performance.

Consistent with previous studies, our results did not reveal any evidence that alterations in body composition and nutrition during RIF could account for the adverse impact on cognitive performance (10, 11, 57, 71). Contrary to a recent meta-analysis suggesting that maintaining training during RIF can lead to a reduction in body mass among adult athletes (72), our results are in agreement with the outcomes of another previous meta-analysis, which indicated that RIF does not have detrimental effects on body composition (73).

### 4.2 Suhoor timing effects

Regarding the Suhoor timing, our findings on cognitive performance reported a notable impact on when Suhoor is consumed, and our findings indicate that having Suhoor later can potentially yield two significant benefits. First, it may ameliorate the decline in performance observed during the afternoon in RIF. Second, it has the potential to maintain midday cognitive performance at levels similar to those experienced before the commencement of Ramadan at the same time of day. We hypothesize that the decline in cognitive abilities during both Suhoor timing conditions, particularly in the afternoon, is primarily due to prolonged fasting periods. Extended fasting can reduce energy availability, affect hormonal and metabolic responses, and lead to dehydration (8).

The observed decline in performance during the afternoon may be attributed to low blood glucose levels, crucial for central nervous system function. With time-restricted feeding, the timing of meals impacts not only glucose but also lipid and hormone levels (74). Following a carbohydrate-rich meal, glucose—the primary energy source—rapidly diminishes due to its utilization for energy and storage as glycogen in the liver. During cognitive tasks, specific brain regions demonstrate increased glucose metabolism (53). Glycogen stores begin to deplete 12–36 h post-carbohydrate intake, triggering fat metabolism and the production of ketone bodies for energy (75). Given that the brain relies on a continuous supply of glucose and cannot synthesize it, this metabolic shift could lead to hormonal changes and fatigue, potentially explaining the afternoon decline in cognitive performance.

However, as the fasting period lengthens throughout the day, energy reserves decrease. This effect varied depending on the timing of fasting between the afternoon fasting duration (~16.5 h vs. 20.5 h) and the midday (~9.5 h vs. 13.5 h) morning fasting duration (~4.5 h vs. 8.5 h) in the S_late_ condition and S_Early_ condition, respectively. Only morning performance under the two conditions remained unchanged because the fasting period did not reach 9 h. However, with extended fasting reaching nearly 20.5 h, performance drops are noticeable, particularly in the afternoon under S_Early_ conditions. At midday, the best performances were observed in the S_late_ condition, which can explain in part the strong effect of the fasting duration related to the last meal intake before starting the fast. While the concept of nutrition timing in the context of sports is relatively recent, recent studies have highlighted the importance of meal timing, quantity, and nutrient composition in sport performance (76, 77). However, importantly, research on how meal timing impacts cognitive performance in athletes, including during Ramadan, is limited, making it difficult to compare our study with others.

Interestingly, higher evening sugar intake and a lengthy gap between meals and sleep correlate with shorter sleep durations (78), potentially revealing the link between meal timing and sleep patterns during Ramadan. Furthermore, changes in daily lifestyle habits, such as increased exposure to nocturnal light and heightened nighttime social activities including late-night shopping, watching TV until dawn, engaging in extended family and friend gatherings, and participating in religious practices like tarawih and fajr prayers may exacerbate sleep pattern disruptions among Muslims during RIF (59, 79). Additionally, the shifting of Ramadan by ~10–12 days earlier each year across seasons (80) has been shown to significantly affect the fasting duration and the seasonal timing of Ramadan. These changes have a notable impact on the health benefits associated with fasting, particularly concerning components of the metabolic syndrome (59, 81). Such variations also potentially alter the frequency, quantity, and timing of Suhoor across different demographic groups characterized by varying ages, and socio-cultural and ethnic backgrounds. A recent systematic review highlighted the significant cultural diversity in food habits among countries that observe Ramadan worldwide (82). However, making comparative conclusions is challenging due to the scarcity of data on Suhoor practices among athletes. These socio-religious and cultural differences must be taken into account when developing beneficial fasting guidelines for diverse populations engaging in RIF.

### 4.3 Strength and limitations

Despite the novelty of our study, several limitations should be acknowledged and addressed in future investigations. A strength of the present study was the evaluation of cognitive performance independently of physical performance at three distinct time points during various periods of RIF in female athletes; however, interpretation of the results should be considered for methodological limitations. First, as we did not evaluate sleep quality or daytime sleepiness using objective measurements (e.g., the Multiple Sleep Latency Test, actigraphy, or polysomnography) in the present study, the data were based only on subjective measurements. Additionally, the study did not consider the participants' menstrual cycle or physiological variables, such as blood glucose levels or hormone levels (e.g., cortisol, adrenaline, melatonin, and noradrenaline). It is crucial to acknowledge that the findings derived our study, focusing on adolescent females, might not be directly transferable to other groups such as older athletes, non-athletic individuals, or males. This is due to variations in physiological fitness and inherent biological differences between genders. Future research should explore the impact of RIF on the diurnal variation of cognitive performance across diverse demographic groups, including male athletes of various ages, disciplines, and ethnic backgrounds. Further studies assessing the effect of RIF on trained and sedentary people could help to further understand the diurnal variation in these cognitive functions during this month. While our study employed a rigorous experimental design with young female athletes, examining a range of confounding factors, such as the time of day, sleep patterns, daily caloric intake, Ramadan fasting, and Suhoor timing, in addition to various cognitive tasks, other potential influencing factors were not monitored. Notably, variables such as the athletes' training load, hydration status, and psychological stress levels could have provided additional insights but were not controlled in our investigation.

## 5 Conclusion

In summary, this study underlines the significance of nutrient timing at different times of day for athletes aiming to enhance their cognitive performance during RIF. The study highlights that when nutrients are consumed, they can substantially influence the diurnal variation in cognitive functions, mainly at noon and in the afternoon. Notably, our findings suggest the advantages of consuming late last meal intake Suhoor for maintaining optimal morning cognitive abilities and avoiding any noon or afternoon impairment during the fasted state, which could affect overall athletic performance.

Nevertheless, additional investigations are required to further understand the ideal nutrient timing approaches tailored to diverse athlete types and sports to achieve performance goals and reduce the risk of injury. This study examines a critical and timely issue concerning millions of athletes worldwide who fast during Ramadan, and understanding how meal timing affects their cognitive performance is crucial for optimizing their training and competition schedules, as cognitive function is integral to athletic success. As a result, athletes and coaches should carefully incorporate nutrient timing into their training and performance strategies. This highlights the importance of aligning meal timing with the body's natural rhythms and daily variations in metabolic and hormonal factors, which is relevant not only to athletes during Ramadan but also to individuals engaged in various types of intermittent fasting and time-restricted eating patterns. The development of exercises and rituals that could improve goal pursuit and cognitive and psychological wellbeing is one prospective application for these studies (83, 84).

## Data availability statement

The raw data supporting the conclusions of this article will be made available by the authors, without undue reservation.

## Ethics statement

The studies involving humans were approved by Local Research Ethics Committee of the University of Jendouba. The studies were conducted in accordance with the local legislation and institutional requirements. Written informed consent for participation in this study was provided by the participants' legal guardians/next of kin.

## Author contributions

HB: Conceptualization, Data curation, Formal analysis, Investigation, Methodology, Validation, Visualization, Writing—original draft. AA: Conceptualization, Funding acquisition, Methodology, Project administration, Resources, Supervision, Validation, Visualization, Writing—review & editing. KT: Validation, Visualization, Writing—review & editing. AB: Investigation, Validation, Writing—review & editing. AS: Data curation, Formal analysis, Validation, Writing—review & editing. HC: Validation, Visualization, Writing—review & editing. HJ: Validation, Visualization, Writing—review & editing. NS: Conceptualization, Investigation, Project administration, Supervision, Validation, Visualization, Writing—review & editing.

## Footnotes

^1^https://data.bnf.fr/fr/11864527/institut_national_de_la_statistique_tunisie/?fbclid=IwAR1499neeV572ADx3ZXqxsK46vY4KL9hPnJZQ8cBV3HPJPFt6NgzJlw3U, (accessed January 17, 2022).
